# Rapid label-free identification of mixed bacterial infections by surface plasmon resonance

**DOI:** 10.1186/1479-5876-9-85

**Published:** 2011-06-07

**Authors:** Jue Wang, Yang Luo, Bo Zhang, Ming Chen, Junfu Huang, Kejun Zhang, Weiyin Gao, Weiling Fu, Tianlun Jiang, Pu Liao

**Affiliations:** 1 Department of Laboratory Medicine, Southwest Hospital, the Third Military Medical University, Chong Qing 400038, P.R China; 2 Department of Laboratory Medicine, Daping Hospital, the Third Military Medical University, Chong Qing 400040, P.R China; 3 Department of Transfusion Medicine, Southwest Hospital, the Third Military Medical University, Chong Qing 400038, P.R China; 4 Chongqing Center of Clinical Laboratory, Chong Qing 400014, P.R China

**Keywords:** bacterial infection, biosensor, mixed infection, surface plasmon resonance

## Abstract

**Background:**

Early detection of mixed aerobic-anaerobic infection has been a challenge in clinical practice due to the phenotypic changes in complex environments. Surface plasmon resonance (SPR) biosensor is widely used to detect DNA-DNA interaction and offers a sensitive and label-free approach in DNA research.

**Methods:**

In this study, we developed a single-stranded DNA (ssDNA) amplification technique and modified the traditional SPR detection system for rapid and simultaneous detection of mixed infections of four pathogenic microorganisms (*Pseudomonas aeruginosa*, *Staphylococcus aureus*, *Clostridium tetani *and *Clostridium perfringens*).

**Results:**

We constructed the circulation detection well to increase the sensitivity and the tandem probe arrays to reduce the non-specific hybridization. The use of 16S rDNA universal primers ensured the amplification of four target nucleic acid sequences simultaneously, and further electrophoresis and sequencing confirmed the high efficiency of this amplification method. No significant signals were detected during the single-base mismatch or non-specific probe hybridization (*P *< 0.05). The calibration curves of amplification products of four bacteria had good linearity from 0.1 nM to 100 nM, with all R^2 ^values of >0.99. The lowest detection limits were 0.03 nM for *P. aeruginosa*, 0.02 nM for *S. aureus*, 0.01 nM for *C. tetani *and 0.02 nM for *C. perfringens*. The SPR biosensor had the same detection rate as the traditional culture method (*P *< 0.05). In addition, the quantification of PCR products can be completed within 15 min, and excellent regeneration greatly reduces the cost for detection.

**Conclusions:**

Our method can rapidly and accurately identify the mixed aerobic-anaerobic infection, providing a reliable alternative to bacterial culture for rapid bacteria detection.

## Background

Anaerobic bacterial infection is one of the major causes of death due to the difficulty to identify the bacteria [1,2]. Among deadly bacteria, *Clostridium tetani *and *Clostridium perfringens *frequently lead to severe infections during wartime and other catastrophes. Mixed aerobic-anaerobic infections, such as infection by *Pseudomonas aeruginosa *and *Staphylococcus aureus*, are frequently undetected and more severe than either single infection [3]. Early and accurate identification of the pathogenic microorganisms in a co-infection is critical for optimizing the treatment, improving the prognosis and decreasing the mortality.

Traditionally, the identification of pathogenic microorganisms mainly depends on a combination of bacterial culture, morphology, biochemical presentations, and immunological examination. Although bacterial culture is extremely time-consuming, it has been the gold standard for identifying bacteria for many years. The growth of anaerobic bacteria always requires rigorous culture conditions, and their phenotypic characteristics (e.g., antibiotic sensitivity and biochemical characteristics) are usually unstable and liable to be affected by gene regulation and plasmid loss [4]. Molecular biological techniques have been widely used to diagnose infections due to their accuracy, rapidity, and specificity. Moreover, nucleic acid amplification by polymerase chain reaction (PCR) allows the detection of trace amounts of target molecules [5,6]. Fluorescent quantitative PCR cannot simultaneously discriminate bacteria in mixed infections, despite its potential for relatively accurate quantification. Electrophoresis is a simple and fast technique, but only semi-quantitative due to its limited resolution. Moreover, discrimination among amplification products with similar lengths using electrophoresis is difficult [7].

Surface plasmon resonance (SPR) provides a highly sensitive method for the detection of biomolecular interactions in a label-free manner. Numerous studies on biomolecular interactions have been conducted with SPR on surfaces coated with a variety of biomolecules, including DNA, RNA, proteins and peptides [8-11]. In previous studies, we successfully constructed a series of gene biosensors based on the quartz crystal microbalance, which was then used to quantify the urine proteins, tumor markers, hepatitis B virus, and human papilloma virus [12-14].

In the present study, we developed a new method using the multi-channel SPR biosensor to rapidly and accurately discriminate the mixed aerobic-anaerobic infection in clinical practice. In this study, DNA from four pathogenic microorganisms (*P. aeruginosa*, *S. aureus*, *C. tetani *and *C. perfringens*) was extracted and amplified simultaneously using universal primers. Single-stranded amplicons were then hybridized with a thiolic probe immobilized on the surface of a multi-channel SPR biosensor. The results were then quantitatively analyzed using an image analysis software. The sensitivity, specificity and reproducibility of this method were also evaluated.

## Materials and Methods

### Materials and reagents

Standard bacterial strains (*S. aureus *ATCC 25923, *P. aeruginosa *ATCC 27853, *C. perfringens *ATCC 64711, and *C. tetani *ATCC 64041) were purchased from the National Institute for the Control of Pharmaceutical and Biological Products, China. Absolute ethanol (analytically pure) was purchased from Chongqing Chemical Reagent Company, China. Lysozyme, proteinase K and bacterial genomic DNA extraction kits were purchased from Qiagen (Germany). dNTPs (0.5 mM for each), 10 × PCR buffer, MgCl_2 _(2.5 mM) and Taq polymerase (5 U/μl) were purchased from Promega, USA. SYBR Green was purchased from DBI, USA. The 16S rDNA Bacterial Identification PCR Kit was purchased from TaKaRa, Japan.

### Main instruments

The following instruments were used: PCR amplifer (GeneAmp PCR System 2400; Perkin Elmer), UV spectrophotometer (Bio-Rad SmartspecTM3000), ABI Prism 310 Genetic Analyzer (PerkinElmer), high-speed centrifuge (Beckman Microfuge 22R), BIO-CAPT gel imaging system (VILBER LOURMAT, BIO-PKOFIL Company, France), electrical thermostatic water bath tank (SHHW21600-II, Yuejing Medical, China), API biochemical identification system and Model FX-DY-252 electrophoresis apparatus (Fuxing Tech, China).

### SPR biosensor

**Figure 1:**
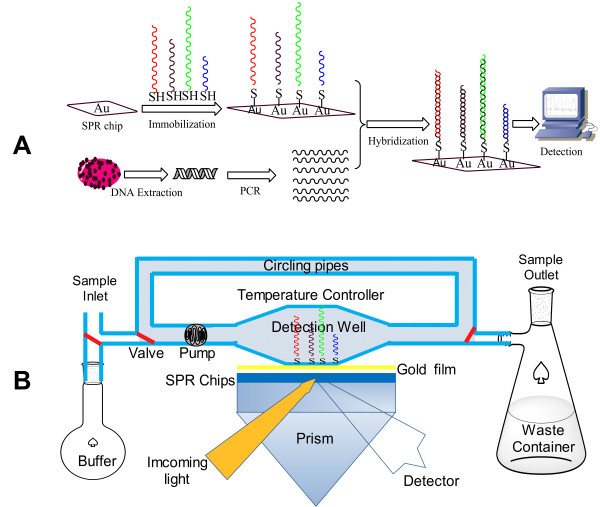
**Schematic diagram of detection with SPR biosensor**. A) The whole detection procedures include probe immobilization, target nucleic acid extraction and amplification, and detection with SPR biosensor. B) The scheme of SPR detection

The SPR biosensor system was modified by our laboratory and composed of an incident light source (polarized light), a sample-loading chamber, a detection well, a temperature control system and a light detector (Figure 1). The sample-loading chamber was designed based on an aspiration mechanism and can suck samples into the detection system through a micro-flow pump. The detection well was designed as a closed, cycle, thin and flat chamber to maximize the contact area in the reaction. A sensor chip (5 mm × 10 mm) with immobilized specific nucleic acid probes was placed in the middle of the detection well. The four probes specific to *S. aureus*, *P. aeruginosa*, *C. perfringens*, and *C. tetani *were arrayed in different zones of the chip surface. The temperature control system was designed based on pulse mechanism and can maintain a predetermined temperature with an accuracy of ±0.1°C at 25~60°C, allowing most nucleic acid hybridizations. The light source system was made up of an incident light source and a signal detector.

The detection principles are as follows: i) when the sample solution contacts the SPR biosensor, the biological molecules bind specifically to the target molecules in the sample solution to form complexes; ii) these complexes may change the surface structure of the biological molecule monolayer, leading to an SPR angle shift; iii) the angle shift is then detected by an optical recording device; and iv) the concentration of target molecule is determined by comparing the resulting angle shift with that in a calibration curve.

### Design of primers and probes

Universal primers (Table 1) were used for the amplification of ssDNA of *S. aureus*, *P. aeruginosa*, *C. tetani *and *C. perfringens*. Four pathogenic bacterium-specific probes were also designed using the Primer Premier Software. To verify the specificity of each probe, three additional nucleotide sequences were designed. Each contained a single-base mismatch to the *S. aureus *probe with one at the 5' end, one at the 3' end and one in the middle of the probe (Table 1). All primers and probes were synthesized by Shanghai BioAsia Company, and the probes labeled with a hydrosulfide at the 5' end.

**Table 1:** Nucleotide sequences of ssDNA used in this study

	Primer a	5'-GTAGGAGTCTGGACCGTGTC-3'
PCR Primers	Primer b	5'-CGGCGTGCCTAATACATG-3'
	Primer c	5'-cgccccGTAGGAGTCTGGACCGTGTC-3'
	*S. aureus*	5'-SH-ACAGCAAGACCGTCTTTCACTTTTG-3'
Probes	*P. aeruginosus*	5'-SH-CCACTTTCTCCCTCAGGACGTATG-3'
	*C. tetanus*	5'-SH-GCCCATCTCAAAGCAGATTACTC-3'
	*C. perfringens*	5'-SH-ATCTCATAGCGGATTGCTCCTTTGG-3'

Single-base	*S. aureus 1*	5'-***T***CAGCAAGACCGTCTTTCACTTTTG-3'
Mismatch sequence	*S. aureus 2*	5'-ACAGCAAGACCG***A***CTTTCACTTTTG-3'
probe	*S. aureus 3*	5'-ACAGCAAGACCGTCTTTCACTTTT***C***-3'

### Bacterial culture and identification

Four lyophilized bacterial strains were cultured in TH broth with sulfate acetate at 37°C for 24 h and then on blood agar plates at 37°C for 24 h. *C. tetani *and *C. perfringens *were inoculated onto the anaerobic blood agar plates and cultured in an anaerobic incubator at 37°C for 48 h. The colonies were selected for microscopic examination and biochemical identification using the API biochemical identification system. API Staph (BioMerieux, USA) was used for identification of *S aureus *and API 20 A (BioMerieux, USA) for identification of *P. aeruginosa*, *C. tetani *and *C. perfringens*..

### Preparation of bacterial DNA

Bacteria suspension was prepared at a density of 1 × 10^8 ^cfu/ml with 0.9% sterile normal saline. Then, 1 ml of bacterial suspension was centrifuged at 8,000 rpm for 5 min at 4°C, and the supernatant was removed. After addition of 10 μl of lysozyme (100 mg/ml), the suspension was incubated at 37°C for 100 min, followed by centrifugation at 4,000 g and removal of supernatant. According to the manufacturer's instructions (FlexiGene DNA Kit, Qiagen, Germany), 400 μl of the eluent were obtained and stored at -20°C for use.

### Amplification of single-stranded DNA and sequencing of four bacterial genes

The mixture for PCR was as follows: 5 μl of 10 × PCR buffer, 4 μl of 10 mmol/l dNTP mix; 1 μl of 10 μmol/l 16s-a, 1 μl of 10 μmol/l 16s-b, 1 μl of 10 μmol/l 16s-c, 0.5 μl of Tap polymerase, 1 μl of template and 36.5 μl of dd H_2_O. PCR was carried out according to the linear-after-the-exponential (LATE)-PCR protocol with slight modification [15]: pre-denaturation at 94°C for 10 min, then 25 cycles of denaturation at 94°C for 30 s, annealing at 49°C for 40 s and extension at 72°C for 40 s, and 40 cycles of denaturation at 94°C for 30 s, annealing at 68°C for 40 s, and extension at 72°C for 40 s and a final extension 72°C for 4.5 min. The PCR products were subjected to 1% agarose gel electrophoresis and visualized using SYBR Green. All PCR products were gel-purified and submitted for sequencing.

### Immobilization of probes onto the biosensor

The reaction was carried out at 45°C using HBS-EP (pH 7.4) as system buffer. The target probes (0.20 μM) were dissolved in HBS-EP (pH 7.4), and 300 μL of this solution was transferred into the detection pipe at a speed of 5 μL/min. A total of 300 μL of HBS-EP (pH 7.4) containing negative control probe (0.20 μM) was transferred into the control pipe at a speed of 5 μL/min. After the reaction completed, the chip surface (precoated with probes) was regenerated by washing with 100 μL of 0.01% SDS and 100 μL of 5 mM HCl at a speed of 50 μL/min. To equilibrate the chip surface, system buffer was supplemented at a speed of 200 μL/min for 30 min.

### Detection of bacteria

The PCR products were added into the SPR monitoring system, and the temperature was adjusted to 45°C. Any change in the refraction angle due to the nucleic acid hybridization was recorded in a real time manner and then converted into electrical signals which were then used to determine the concentration using the system software.

### Calibration

DNA was extracted from each standardized bacterial strain (50 cfu/ml) and subjected to amplification by PCR according to procedures described above. The products were diluted to 100, 50, 10, 5 and 1 nM and then hybridized with the specific probes on the SPR biosensor. Finally, standard curves were delineated.

### Determination of sensitivity

The buffer without bacteria was added to the detection well as a blank. The blank was tested 10 times, and the average and three standard deviations were used as the baseline detection limit.

### Determination of probe specificity

After each *S. aureus *probe (1 μM) and the single-base mismatch sequence probes (*S. aureus *1, 2 and 3) were immobilized on the surface of SPR biosensor, the PCR product (100 nM) of *S. aureus *was added to the detection well. The changes in the refraction angle due to nonspecific binding were recorded. Then, the probes specific for four bacteria were immobilized on the SPR chips. The product of a combined four-bacterium pure culture was added to the detection well, and the changes in the refraction angle due to nonspecific binding were recorded.

### Regeneration performance testing

After each detection, 100 μL of 0.01% SDS and 100 μL of 5 mM HCl were added to the detection well to dissociate the bound target DNA. Then, the well was washed thrice with PBS. The same sample was re-added to the well, and the hybridization signal recorded. The concentration of samples was 50 nM and this procedure was repeated 200 times to determine the regeneration performance.

### Clinical sample detection

DNA was extracted from 365 tissues infected with *S. aureus*, *P. aeruginosa*, *C. tetani *and *C. perfringens *(as confirmed by bacterial culture). All experiments were performed with the approval of the Ethics Committee of Third Military Medical University. After amplification by PCR, the resulting products were added to the SPR detection well as described above. Then, the positive and negative detection rates were determined.

### Data analysis

All experiments were performed at least three times and statistical analysis was performed with SPSS version 15.0 (Statistical Package for the Social Sciences, SPSS Inc, Chicago, Illinois). The changes in SPR angle were presented as the means ± standard deviation (SD). One-way analysis of variance (ANOVA) was used to compare the differences among different probe groups. McNemar's test was employed to compare the consistency between the SPR detection and the traditional culture method. A value of P < 0.05 was considered statistically significant.

## Results

### Bacterial culture and isolation

Colonies obtained by bacterial revival, isolation and culture were identified using the API biochemical identification system and used as the target bacterial strains (data not shown).

### Identification of PCR products

**Figure 2:**
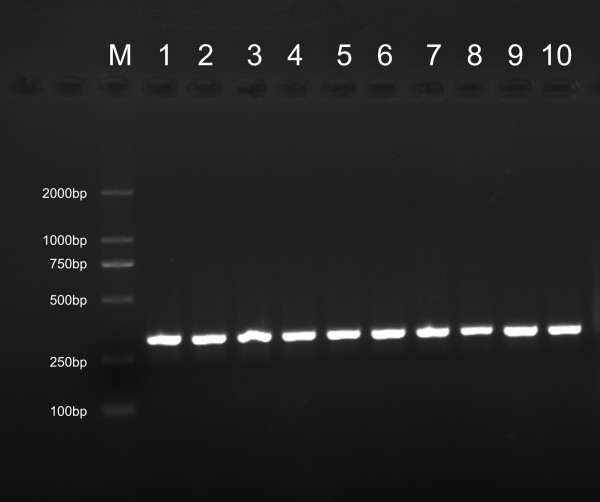
**Electrophoresis of single-stranded PCR products**. All the nucleic acids were stained by SYBR Green II. The marker was lightly stained, whereas the optical density of ssDNA band was relatively high. Lane 1 and 2: *C. perfringens*, lane 3 and 4: *C. tetani*, lane 5 and 6: *P. aeruginosa*, and lane 7 and 8: *S. aureus *in duplicates. Plasmid of *P. aeruginosa *(lane 9), and *S. aureus *(lane 10) were used to identify the length of ssDNA.

Although the marker was understained (lane M), the PCR products in lanes 1 to 8 were bright (Figure 2). In addition, the *P. aeruginosa *(lane 9) and *S. aureus *(lane 10) plasmids had similar brightness and position as the PCR products, indicating that most of PCR products were ssDNA. Sequencing confirmed that the four specific sequences after PCR amplification were the expected sequences of *S. aureus*, *P. aeruginosa*, *C. tetani *and *C. perfringens *(data not shown).

### Specificity of the detection with SPR biosensor

**Figure 3:**
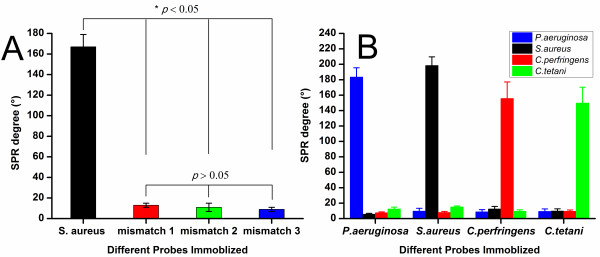
**Specificity of the detection with SPR biosensor**. A) Hybridization of PCR products of *S. aureus*. A total of 50 nM of the PCR products of *S. aureus *were incubated with four different probes: a specific probe (black column), a 5'-end single-base mismatch probe (red column); a middle single-base mismatch probe (green column); a 3'-end single-base mismatch probe (blue column). * *P *< 0.05 vs other three single-base mismatch probes. B) Hybridization of a mixture of PCR products from four bacteria with four specific probes.

Two experiments were designed to validate the specificity of the detection with SPR biosensor. In the presence of a complementary sequence with a single-base mismatch, the change in the SPR angle was small (Figure 3A), and there was no significant difference among the SPR angle shifts for the three different probes with mismatch in different sites. Cross-reaction between the target and the non-specific complementary probes was very low (Figure 3B).

### Calibration and baseline detection limit

**Figure 4:**
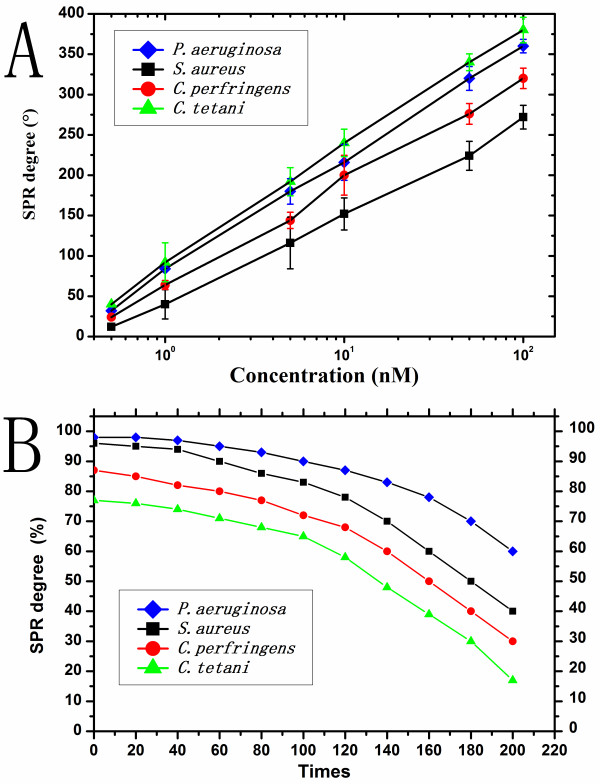
**A) Calibration curves of each bacterium at the concentrations of 0.1 nM, 0.5 nM, 1 nM, 5 nM, 10 nM, 50 nM and 100 nM**. All curves fitted well logarithmically, with the formulas as follows: y = 0.153 × ln(x) + 0.197, with R^2 ^= 0.9991 for *P. aeruginosa *(blue diamonds); y = 0.121 × ln(x) + 0.103, with R^2 ^= 0.9974 for *S. aureus *(black squares); y = 0.139 × ln(x) + 0.157, with R^2 ^= 0.9974 for *C. perfringens *(red circles); and y = 0.160 × ln(x) + 0.222, with R^2 ^= 0.9994 for *C. tetani *(green triangles). B) Regeneration of the detection with SPR biosensors. Data were expressed as the percentage of maximal SPR degree angle. All the SPR angles decreased with an increase of regeneration. All the SPR angles decreased slightly during the first 100 tests but were still higher than 80%, whereas they dropped rapidly in the another round of 100 tests

Serial dilutions of the PCR products (100, 50, 10, 5, 1, 0.5 and 0.1 nM) were measured to calibrate the detection with SPR biosensor. All the correlation coefficients of the standard curves were >0.99, indicating favorable linearity (Figure 4A). The detection limits were 0.02 nM for *S. aureus*, 0.03 nM for *P. aeruginosa*, 0.03 nM for *C. perfringens*, and 0.01 nM for *C. tetani*.

### Detection of clinical samples

Among 365 samples, all were found to be infected by one or more of these four bacteria demonstrated by a culture-based method. The sensitivity and specificity of the detection with SPR biosensor were 92.86% and 95.65%, respectively, for *P. aeruginosa*, 98.33% and 100%, respectively, for *S. aureus*, 96.67% and 97.14%, respectively, for *C. perfringens *and 91.67% and 96.23%, respectively, for *C. tetani *(Table 2). These findings indicate good consistency between the detection with SPR biosensor and the traditional culture method.

**Table 2:** Comparison of the SPR biosensor and bacterial culture in the detection of four bacteria

	SPR biosensor method								
Culture method	*P. aeruginosa*		*S.aureus*		*C.perfringens*		*C.tetani*		Total
		
	P*	N**	P	N	P	N	P	N	
P	238	3	249	2	228	1	110	2	
N	4	120	1	113	2	134	1	252	
Total	242	123	250	115	230	135	111	254	365

* P: positive, and **N: negative. No significant difference in the detection of four bacteria in mixed infection was found between bacterial culture and SPR biosensor (*P *> 0.05). The differences and 95% CI were 3.08% and -3.76%~6.08%, respectively, for *P. aeruginosa*; 1.54% and -1.46%~1.45%, respectively, for *S.aureus*; 0% and -3%~3%, respectively, for *C. perfringens *and 1.54% and -3.74%~4.54%, respectively, for *C.tetani*.

### Regeneration performance

Results demonstrated that the detection with SPR biosensor had good regeneration performance. Over the first 100 regeneration tests, the SPR angle decreased < 20%. After 100 regeneration tests, however, the hybridization efficiency decreased rapidly. After 200 regeneration tests, the efficiency was <50%. These findings indicate that a well-immobilized SPR biosensor chip can be regenerated more than 100 times (Figure 4B).

## Discussion

Discriminating a mixed bacterial infection by traditional culture- and biochemical character-based methods is a challenge in clinical practice because the bacteria in the mixed infection are apt to produce atypical phenotypes. Molecular biological methods such as SPR biosensing can detect the specific nucleic acid of bacterial genomes and thus avoid the difficulties associated with phenotypic changes. Currently, the 16S rDNA, a gene encoding the small ribosomal RNA subunit, is widely used for the identification of bacteria in the mixed infection because its sequence contains conserved regions common to all bacteria and divergent regions unique to each species. Although amplification using universal primers is critical for the multiple target analysis, it usually leads to nonspecific PCR products [16]. In this study, the formation of nonspecific PCR products was avoided by optimizing the PCR reaction conditions. Sequencing showed that the universal primers successfully amplified the target DNA from all four bacteria in the analyte mixture.

Amplification of single-stranded DNA is a notable characteristic of this method. Conventional PCR usually consists of 35 cycles of reaction and yields double-stranded products that require being unwound at high-temperature before they can be detected through hybridization. This may correspondingly increases the number of steps and the complexity of device. In addition, it often leads to incomplete unwinding or mismatches between some bases, which is inconvenient for the development of a specific and sensitive assay. Therefore, single-stranded DNA was used for hybridization. According to the LATE-PCR protocol and previously reported [17], we designed three universal primers to ensure the formation of ssDNA. Electrophoresis showed that most of the products were ssDNA. Sequencing confirmed that the amplified ssDNA was the target sequence, indicating that this method accurately amplified ssDNA.

**Figure 5:**
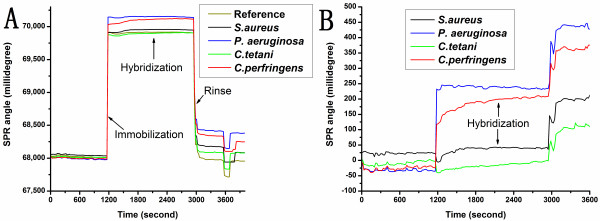
**Real-time detection of four bacteria by SPR biosensor**. A) Real-time detection of four bacteria. B) Each detection included immobilization, hybridization and rinsing. Red line: *S. aureus*, blue line: *P. aeruginosa*, cyan line: *C. tetani*, pink line: *C. perfringens*. The difference in the SPR angle between the pre-hybridization stable phase and the post-hybridization stable phase is the angle shift induced by specific hybridization.

SPR systems are sensitive to the changes in the thickness or refractive index of the gold film coated at the interface between the chip surface and an ambient medium. Hybridization between a probe immobilized on the chip surface and its target may cause the conformational changes in the surface of the gold electrodes leading to corresponding changes in the refractive index. SPR has several advantages in clinical practice. Firstly, it has the capability of real-time monitoring, which is a crucial characteristic of biosensors and also reduces the detection time. Once the refractive index changes when the DNA-DNA reactions between the probes and target sequences occur, hybridization can be detected in a real-time manner by continuously monitoring the refractive index of the gold film coated on the sensor (Figure 5). Secondly, this method is a label-free technique. Thus, the problems associated with fluorescence quenching or radioactive exposure are avoided. This technique also improves the accuracy of detection and reduce the detection time [18].

Rapidity is the most prominent advantage of this method. This detection can be finished within 15 min, and the whole detection process, including DNA extraction, denaturation, PCR amplification and real-time detection, can be done within 3~4 h. In addition, the conventional DNA extraction and denaturation were employed into this method because both techniques are mature and commercially available.

To increase the accuracy of detection, four probes were arranged in a tandem model, and samples containing mixed bacteria passed through the detection well to hybridize with probes specific for *S. aureus*, *P. aeruginosa*, *C. tetani *and *C. perfringens*. At the optimal temperature, specific nucleic acid probes hybridized with their specific target sequences, which gradually decreased the amount of target molecules in the sample. To increase the accuracy of detecting low-concentration analytes, the samples repeatedly passed through the tandemly arranged probes in a circulating detection well. The reaction time could be controlled by adjusting the flow velocity, and the optimal velocity was determined to be 3~5 mm/s. The advantages of a tandem probe array include the high accuracy, the low interference between probes, and the possibility of simultaneous detection of more target molecules by simply increasing the types of tandem probes.

The sensitivity and specificity are crucial determinants of sensor performance, which were also investigated in this study. The results demonstrated that this method had a sensitivity equivalent to conventional culture method. The analysis of specificity demonstrated that hybridization did not occur in the probes containing single-base mismatches. The location of the mismatch site within the probe did not affect the results, which was partially consistent with previously reported [19,20]. This may be attributed to that the SPR angle shifts induced by all three types of hybridization were too low to be discriminated by the biosensor. There were no obvious cross-reactions between the four bacteria (Figure 3). These findings demonstrate the high efficiency of SPR biosensor. Testing clinical samples indicated that this method and the traditional culture method correlated significantly in terms of the detection rate. Our method, however, can shorten the detection time substantially from one week in traditional method to 2~3 h.

Although this biosensor successfully identified different types of microorganisms in most clinical samples, it is currently unable to quantify the bacterial load *in vivo*, which is important for clinical assessment, medication and prognosis. Because this method involves PCR amplification, quantitative analysis relies on the quantity of the template during the pretreatment, and multiple factors may affect the outcome of this analysis. A standardized sample processing procedure is therefore required to accurately quantify these pathogenic bacteria.

## Conclusions

Our method allows for the simultaneous, real-time discrimination of *S. aureus*, *P. aeruginosa*, *C. tetani *and *C. perfringens *in mixed bacterial infections. Moreover, this method has a specificity equivalent to bacterial culture-based methods and allows for the semi-quantitative assessment of multiple bacteria, which is helpful for the clinical diagnosis and follow-up treatment. This method may become a highly promising technique for the microorganism analysis.

## List of abbreviations

SPR: Surface plasmon resonance.

## Competing interests

The authors declare that they have no competing interests.

## Authors' contributions

JW and YL have made substantial contributions to conception and design, data acquisition, analysis and data interpretation and are involved in drafting and revising the manuscript. WF has made substantial contributions to conception and design.

BZ, MC, TJ, PL, JH, KZ and WG have made substantial contributions to data acquisition, analysis and data interpretation. Moreover, each author has taken public responsibility for appropriate portions of the content. All authors read and approved the final manuscript
